# Biodiversity of Entomopathogenic Fungi in the Soils of South China

**DOI:** 10.3390/microorganisms7090311

**Published:** 2019-09-03

**Authors:** Xiaoyan Niu, Weiwen Xie, Jing Zhang, Qiongbo Hu

**Affiliations:** Key Laboratory of Bio-Pesticide Innovation and Application of Guangdong Province, College of Agriculture, South China Agricultural University, Guangzhou 510642, China (X.N.) (W.X.) (J.Z.)

**Keywords:** entomopathogenic fungi, soil, biodiversity, bioactivity, whitefly

## Abstract

The southern part of China, located in tropical and south subtropical areas has unique natural environments, but the distributions of entomopathogenic fungi (EFs) in the soil are not clear. In this research, 198 soil samples were collected from the four Provinces (Autonomous Region) of South China. The results indicated that a total of 292 fungal isolates were obtained from 176 soil samples. Then, based on the morphological and rDNA-ITS sequences analysis, 213 EFs isolates of 19 species in 12 genera were identified. Furthermore, *Purpureocillium lilacinum* with 75 isolates was recognized as the absolutely dominant EF species, while *Isaria javanica*, *Metarhizium anisopliae*, and *Beauveria bassiana* (respectively with 29, 26, and 26 isolates) were the richer species. The data also indicated that Guangxi Province has the best EFs diversity with the Shannon–Wiener index (SWI) of 2.29, the soils covered with grass had the best EFs diversity with the 2.14 SWI, while the orchard and fallow land had the lowest SWI of 1.52, which suggested that the diversity of plants and insects on ground, as well as the massive application of broad-spectrum fungicides, affect the EFs diversity in the soil. Finally, the rare species, *Nectria mauritiicola* and *Scopulariopsis brumptii* were first reported about their entomopathogenic activities against *Bemisia tabaci*. Our experiment will give new insights to the understanding of EFs distribution characteristics and their biodiversity conservation.

## 1. Introduction

Entomopathogenic fungi (EFs), a kind of important pathogens infecting host insects (arthropods), play key roles in the regulation of insect populations and biotransformation in natural biosystems. Differing from the bacteria and virus pathogens infecting insects through mouthparts and guts, EFs invade insects through the cuticles [1]. The popular EFs, *Beauveria bassiana*, *Metarhizium anisopliae*, *Purpureocillium lilacinum*, and *Isaria fumosorosea*, etc., have been developing as mycopesticides to control agricultural, forest, and disease vectors pests such as locusts, grubs, aphids, whiteflies, moths, mosquitoes, and phytopathogenic nematodes, etc. [2,3]. On the other hand, a lot of EFs such as *Cordyceps* spp. are expensive edible and medical mushrooms and have been used for traditional health foods and medicines in East Asia [4,5]. Additionally, EFs produce multiple secondary metabolites with some bioactivities and functions, which have the potencies for medicines or nutriments [6]. However, according to the report of ‘State of the World’s Fungi 2018′, the natural EFs resources are declining, for example, the annual yield of the Chinese caterpillar fungus (*Ophiocordyceps sinensis*) had declined from more than 100 tonnes in the 1950s to 5–15 tonnes in the 1990s [7]. Obviously, it is urgent to conserve the EFs resources.

Most EFs are soil-dwelled microbes. Soil provides a destination for insect fungal diseases and a surrounding for the EFs rhizosphere, but the soil conditions such as the temperatures, moistures, pHs, and microbes influence the EFs persistence and survival [8,9,10]. For example, *B. bassiana*, *M. anisopliae*, and *M. pingshaense* can persist and survive in soil for long times, but they are closely affected by nutritional, microbial, and physiochemical factors of the soil. Additionally, in fact, EFs have proliferation in the soil with enough nutrition [11,12,13,14]. Moreover, the residues of pesticides and heavy metals affect the soil microbial activities and fungal community structures [15,16]. However, the statuses of EFs in soil stages are not well understood by researchers. Investigating the soil EFs will be beneficial to the discovery of new species and conservation of EFs resources [17,18]. Meanwhile, in order to better recognize the mechanisms of occurrences and the storage of EFs, it is necessary to investigate the soil EFs [19].

South China is located in the tropic and south subtropical areas with hot, moisture, rainy climatic, acidic soils and multiple vegetation, which is a center with abundant biodiversity [20]. Moreover, South China is a district of unbalanced economic development, for example, the areas of Pearl River Delta belong to the developed industrial district having a heavier environmental pollution and ecological safety of exotic species, while the remote mountainous areas of Guangxi, Guizhou, and Yunnan Provinces belong to the under-developing or poor regions with better natural environments [21]. However, the distribution of soil EFs in these areas are not clear. Therefore, the purpose of this research is to investigate the EFs distribution and abundance in different soil habits in South China areas including the provinces (Autonomous Region) of Fujian, Guangdong, Hainan, Guangxi, Yunnan, and Guizhou. Then, it is intended to analyze and discuss the influences of human activities and environment changes on the EFs. The research will give some new insights to the EFs biodiversity and their distribution characterization.

## 2. Materials and Methods

### 2.1. Soil Sample Collection

**Figure 1:**
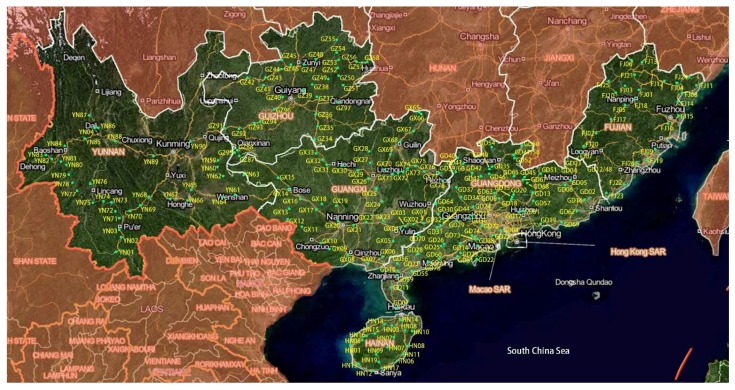
The map of sites distribution for the soil samples collection.

The soil samples were collected from the location sites with various types of covered plants including crop lands (farming lands covered with crops such as grains, tomatoes, vegetables, etc.), fallow lands (farming lands but no crops growing), forests (the lands covered with forests except eucalyptus trees), grasses (the lands covered with grasses) and eucalyptus forests (the lands covered with eucalyptus trees). The longitude and latitude in each site were recorded by ICEGPS 100C (Shenzhen, China). From each site, approximately 200 g of soil beneath the ground 10–15 cm in three randomly selected points were collected and mixed as a sample stored in a plastic bag at 4 °C for further use. The total 198 samples were collected from 170 sites in four provinces including Fujian, Guangdong, Guangxi, and Hainan in South China (Figure 1).

### 2.2. Isolation of Fungal Species from the Soil Samples

The method of our previous experiment [8] was referred to the isolate fungal strains from the soil samples. Totally, the samples were pre-treated to remove stones and plants residues, then the soil suspensions of 0.02 g/mL were prepared with the 0.1% Tween-80 solution. The 0.1 mL suspension was inoculated onto a selective medium plate (when PDA 1000 mL was cooled to 50 °C, 0.2 g cycloheximide, 0.2 g chloramphenicol, and 0.0133 g rose Bengal sodium were added) and the fungal colonies were picked out to PDA plates for culture and further uses [22]. Each sample was subjected to fungal isolation two times.

### 2.3. Identification of Fungal Species and Analysis of Genetic Homology

The identification of fungal isolates was based on the morphological characteristics and similarity of the rDNA-ITS sequences. The method of our previous experiment [8] was referred. In summary, an optical microscope system equipped with a digital camera (MC-D500U, Phenix, Jiangxi, China) was employed to carry on the morphological analysis to measure the mycelia, conidia, and sporulation structures of fungal isolates. The DNA extraction kits (DP3112, Bio-Teke, Beijing, China) were used to extract the total DNA from fungal isolates. The primers ITS1 (5′-TCCGTAGGTGAACCTGCGG-3′) and ITS4 (5′-TCCTCCGCTTATTGATATGC-3′) were used to amplify the fungal ITS regions with the standard PCR cycling protocol on the T100^TM^ Thermal Cycler (BIO-RAD, USA). The PCR products were visualized with UV illumination and photographed (Tanon-1600, Tanon, Shanghai, China) and sequenced by Sangon Biotech on an ABI-PRISM3730 automated sequencer (Applied Biosystems, USA). The obtained rDNA-ITS sequences were submitted on a GenBank and compared with similar sequences through the BLAST of NCBI. The phylogenetic trees of the EFs were constructed by MEGA X [23] with a statistical method of maximum likelihood, a bootstrap test of 500 replications, and the Jukes–Cantor model. The standard EF strains were referred (Table 1).

### 2.4. Evaluation of Shannon–Wiener Index

The biodiversities of fungi in different soils were evaluated by the Shannon–Wiener index (SWI). SWIs were calculated based on the formula SWI = −$\sum_{i}^{s}\left(Pi\right)\left(lnPi\right)$, where *s* is the total number of species in the sample, *i* is the total number of individuals in one species, *Pi* is the proportion of species I in the sample, *lnPi* is the value of the natural logarithm of *Pi* [47].

### 2.5. Bioassay of the Fungal Strains on the B-Biotype Whitefly

The isolates of rare fungal species were subject to a bioassay against the B-biotype whitefly (*Bemisia tabaci*) based on the reference [8]. Summarily, the fungal conidia suspensions of 1.0 × 10^8^ spores/mL were prepared with a 0.02% Tween-80 solution. The population of the B-biotype whiteflies used in this study was a greenhouse population reared for >20 generations. *Hibiscus rosa-sinensis* was cultured in cages to feed the insects. The second instar nymphs were selected for the bioassay based on the leaf immersion method (China standard NY/T 1154.1 4-2008). In brief, the *H. rosa-sinensis* leaves with second instar whitefly nymphs were dipped into conidial suspension for 20 s. The pest’s numbers were surveyed each 24 h after treatment. The nymphs were considered as diseased death when they lost their normal yellow-green color, turgidity, and smooth cuticle structure, and subsequently mildew grown. The 0.02% Tween-80 solution was used as a control group. The experiment was replicated three times. The accumulated mortalities (%) were subjected to an analysis of DMRT (Duncan’s multiple range test) by using DPS 9.5 (Data Processing System, Zhejiang, China) [48].

## 3. Results

### 3.1. Entomopathogenic Fungi Species Diversity in Soils of South China

**Figure 2:**
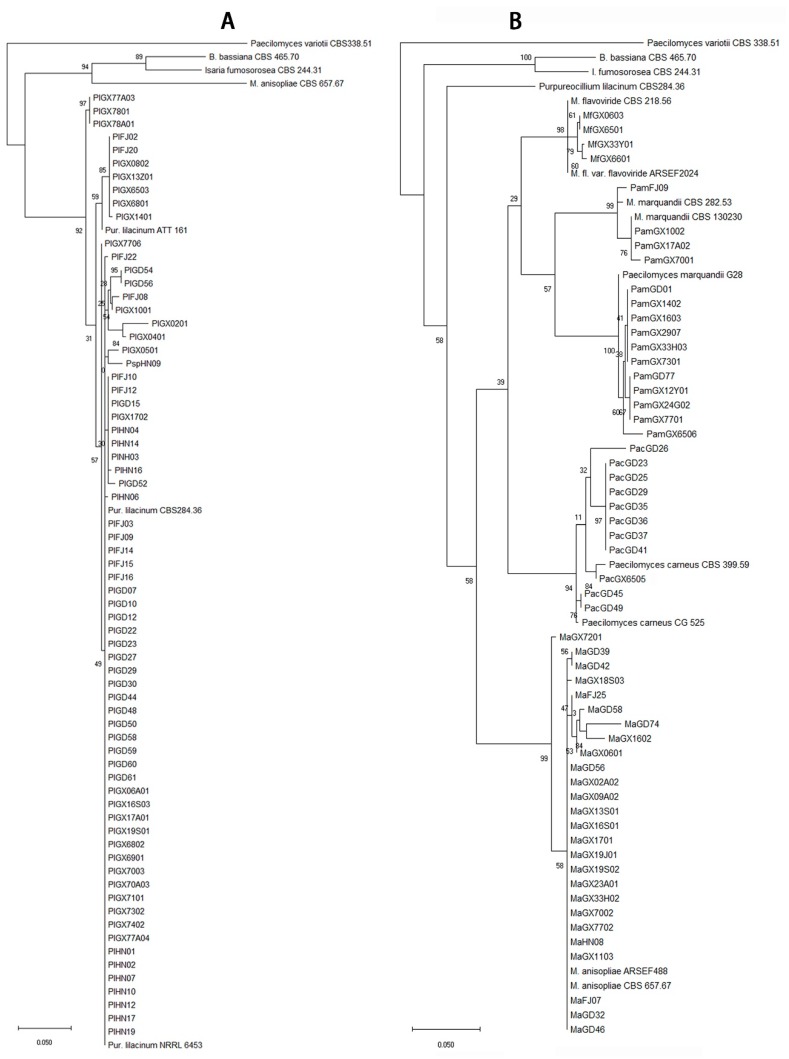
Phylogenetic tree of *Purpureocillium* spp. (**A**) and *Metarhizium* spp. (**B**) isolates.

**Figure 3:**
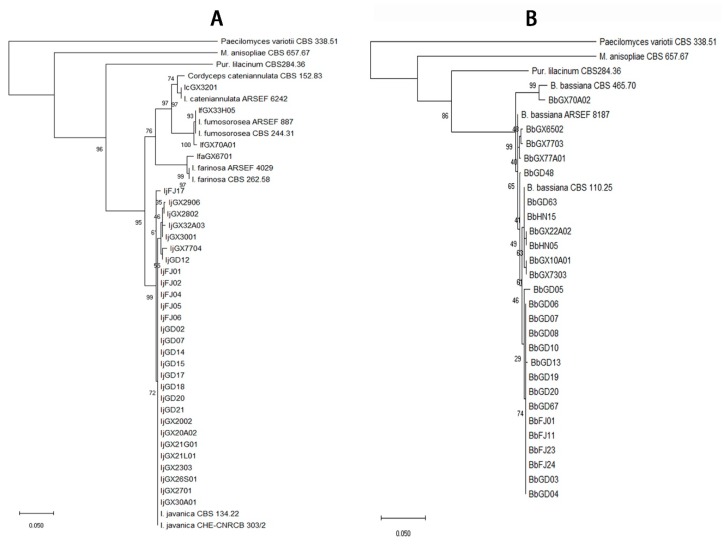
Phylogenetic tree of the *Isaria* spp. (**A**) and *Beauveria bassiana* (**B**) isolates.

**Figure 4:**
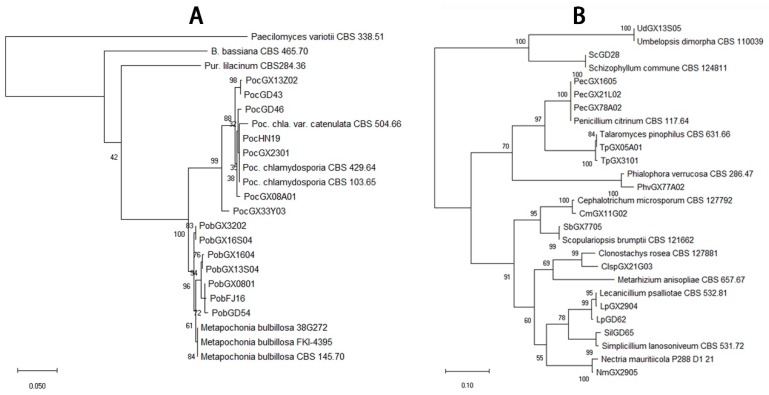
Phylogenetic tree of the *Pochonia/Metapochonia* spp. (**A**) and other (**B**) isolates.

The total of 292 fungal isolates were purified. Among them, 213 EF isolates were identified as 19 species that belong to 12 genera according to the morphological and molecular analysis. *Purpureocillium lilacinum* with 75 isolates was the richest species, but the congeneric species *P. lavendulum* had only two isolates (Figure 2, Table S1). The genus *Metarhizium* had four species, *M. anisopliae*, *M. flavoviride*, *M. marquandii*, and *M. carneum*, which 26, 4, 15 and 11 isolates were respectively obtained (Figure 2, Table S1). The genus *Isaria* had four species including *I. cateniannulata*, *I. farinosa*, *I. fumosorosea*, and *I. javanica* with 1, 1, 2 and 29 isolates (Figure 3, Table S1). The genus *Beauveria* had only one species *B. bassiana* with 26 isolates (Figure 3, Table S1). Both the *Pochonia chlamydosporia* and its close species *Metapochonia bulbillosa* all had 7 isolates (Figure 4, Table S1). Other species with 1–3 isolates were respectively identified as *Nectria mauritiicola*, *Scopulariopsis brumptii*, *Clonostachys* sp., *Lecanicillium psalliotae*, *Phialophora verrucosa*, *Simplicillium lanosoniveum*, *Cephalotrichum microsporum*, *Penicillium citrinum*, *Schizophyllum commune*, *Talaromyces pinophilus*, and *Umbelopsis dimorpha*, in which the last five species have not been entomopathogenic fungi (Figure 4, Table S1). The other 71 isolates were not classified yet. Obviously, *P. lilacinum*, *I. javanica*, *B. bassiana*, and *M. anisopliae* were the most abundant EFs species.

### 3.2. The Distribution of Soil EFs in Different Areas of South China

There were different numbers and isolating rates of EFs in various areas of South China. Compared with the average fungal isolating rates of 88.89% and 75.76% in all the fungi and EFs samples, Hainan had the highest rates of >90% (Table 2). However, the Shannon–Wiener index indicated that Hainan was the lowest EFs biodiversity district, while Guangxi had the best EFs biodiversity with a SWI of 2.29. Guangdong and Fujian had the SWI 1.4–1.9 (Table 2).

### 3.3. The Biodiversity of Soil EFs in Different Samples Environments

There were different numbers and isolating rates of EFs in various areas of South China. Compared with the average fungal isolating rates of 88.89% and 75.76% in all the fungi and EFs samples, grass and orchard samples had the larger fungal rates of >95%, while the crop and orchard samples had higher EFs rates of >80% (Table 3). However, the Shannon–Wiener index indicated that the orchard and fallow land had the lowest EFs biodiversity with the SWI of 1.52, while the grass samples had the best EFs biodiversity with a SWI of 2.14. Crop, eucalyptus, and forest had the SWI 1.8–2.0 (Table 3).

### 3.4. The Pathogenicities of Fungal Isolates against B-Biotype Whitefly

**Figure 5:**
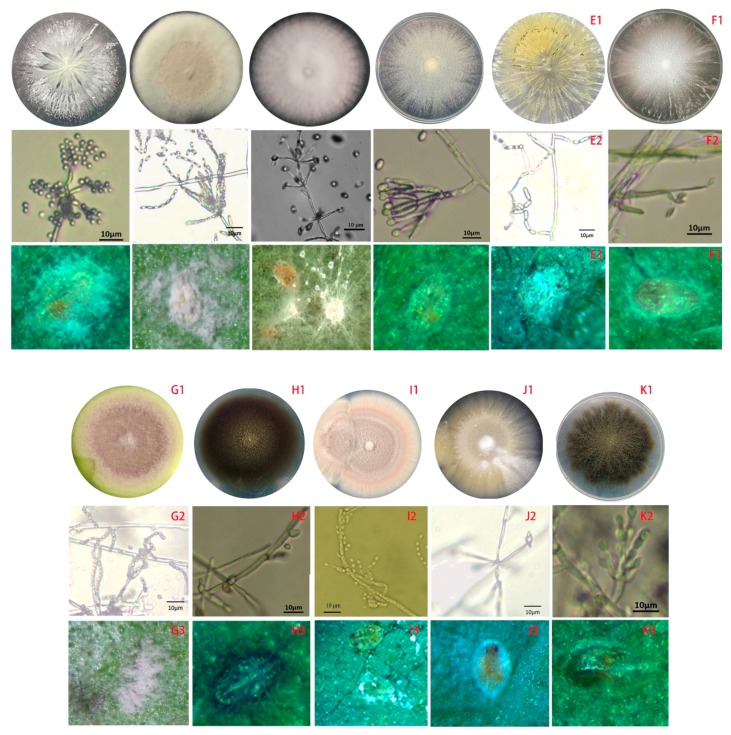
The feature profiles of entomopathogenic fungi (EFs) and the symptoms of whiteflies infected by different isolates. **A**, BbGD07 of *Beauveria bassiana*; **B**, IfFJ05 of *Isaria fumosorosea*; **C**, LpGD62 of *Lecanicillium psalliotae*; **D**, ClspGX21G03 of *Clonostachys* sp.; **E**, MaFJ07 of *Metarhizium anisopliae*; **F**, NmGX2905 of *Nectria mauritiicola*; **G**, PamGD01 of *Metarhizium marquandii*; **H**, PhvGX77A02 of *Phialophora verrucosa*; **I**, PlHN01 of *Purpureocillium lilacinum*; **J**, PocGD43 of *Pochonia chlamydosporia*; **K**, SbGX7705 of *Scopulariopsis brumptii*; 1, colony; 2, conidial structure; 3, symptoms of whiteflies. Bar = 10 μm.

The 15 isolates were subjected to the bioassay against a B-biotype whitefly of second instar nymphs. The results indicated that the four isolates, CmGX11G02 of *Cephalotrichum microsporum*, PecGX1605 of *Penicillium citrinum*, TpGX05A01 of *Talaromyces pinophilus*, and UdGX13S05 of *Umbelopsis dimorpha*, were not effective to the whitefly, because they gave the insignificant mortalities (no different from the control) (*p* < 0.05) to the insect (Table 4). However, the other 11 isolates all had substantial pathogenic activities to the whitefly (Table 4). Additionally, it was first found that *Nectria mauritiicola* and *Scopulariopsis brumptii* had the entomopathogenic activities. In addition, the symptoms of disease insects usually become brown, black colors and not shiny on the surface, finally, the EFs hyphae grown out of the dead body (Figure 5).

## 4. Discussion

It is difficult to compare and evaluate the biodiversities of the soil EFs, due to the lack of general concepts and reliable approaches to define microbial species, studies on the soil microbial communities concentrate on genetic diversity and functional diversity more than the species diversity [49]. To investigate the soil EFs, it is necessary to employ a reliable approach. Traditionally, the *Galleria mellonella* (greater wax moth) bait is a common tool for the EFs isolation from soil. However, it needs more consumption of work and times, while probably some strains (for example, those non-pathogens to the great wax moth) could be not obtained [17,50]. Then, the metagenome has been developing for diversity research of soil microbes in the recent decade [51], it can detect the soil EFs species, but it cannot isolate the EFs strain and discover new EFs species. In addition, selective media with the advantages of more simple and economical technology have been using to separate the soil EFs [52].

Here, we investigated the EFs distribution in a larger scale of South China by using a selective medium. Undoubtedly, our results first provide a great deal of information of the soil EF species and strains in these areas. The author’s group has been using the selective medium to obtain many EFs in recent years [8], we think that many factors influence the EFs isolation in practice. First, it is difficult to isolate the EFs from the too wet soil, because high moisture seriously affects the EFs survival in soil [14]. Second, the stored-stage of the soil sample is very important as well. Generally, the stored-stage of six months at 0 °C does not influence the EFs isolation of the soil samples, but if conserved for a longer time, the EFs in the samples will quickly decrease. In addition, the soil samples numbers or the density of the sample collection changes the statistic results of the EFs numbers in an area. In this study, Hainan with less EF isolates might be related to the smaller soil samples and in the rain collected.

Moreover, the result data indicated that the soil environment (vegetation) closely impacts the EFs distribution. Compared to grass and forest samples, the orchard and fallow land samples had less EFs. It might be because there are poorer species of plants and insects and more farming activity in the orchard and fallow land, which might affect the EFs diversity. In fact, among so many farming factors, the fungicides utilization is probably the most important reason to reduce the EFs in the soil. China is a big pesticides consumer, a large number of broad-spectrum fungicide such as carbendazim, chlorothalonil, cancozeb, and azoxystrobin, etc., were sprayed in fields every year, which probably prohibit fungi in the soil [53,54,55]. However, crop samples had a better EFs diversity than the orchard and fallow land samples, it is maybe related to the cropland that had more plants and insects species, because crops include so many vegetables, grains, etc., but the orchard of South China are mainly Litch, Longan, banana, and mango, etc.

Interestingly, eucalyptus seems not to affect the EFs diversity. Eucalyptus is well-known as an effective reforestation tree species, due to its fast growth and high adaptability to various environmental conditions. However, the introduction of eucalyptus had negative effects such as soil degradation, decline of the ground water level, and decrease of biodiversity [56]. Eucalyptus has a strong allelopathy to inhibit other plants and some soil microorganisms [57], but their effects on the EFs have not been reported yet. The relationship of the EFs and eucalyptus is worthy of further research.

*Purpureocillium* is a new genus constructed from *Paecilomyces* *lilacinus* [44]. *Purpureocillium lilacinum* is a combination with obvious intraspecific diversity. Many strains of the species have been used as biocontrol agents of phytopathogenic nematodes [58,59], while some strains have insecticidal potency [60] and some others have the risks to cause infection in immunocompromised humans [61,62]. In this experiment, *P. lilacinum* is also the most abundant species isolated from whole six regions with the most 75 isolates, in which the differentiation of the species is found from the phylogenetic tree constructed with these isolates. It is believed that more *Purpureocillium* species will be discovered in the future. The prevalence of the *Purpureocillium* fungi in the soil is probably due to intraspecific genetic diversity, extensive uses, and the mass occurrence of the plant root knot nematode disease [63]. However, the similar species, *P. lavendulum* had only two isolates found in this experiment. The reason might be related to the fact that *P. lavendulum* cannot grow at 35 °C [64], which probably influence its distribution in the hot areas of South China.

*B. bassiana* and *M. anisopliae* are famous EFs species that infect a lot of insects. The two EFs have been used as biocontrol agents for a long time, which is maybe beneficial to their accumulation in soil [65,66,67]. Therefore, the two EFs were isolated from whole regions in this experiment. *M. marquandii* and *M. carneus* were originally classified to the genus *Paecilomyces*, they were often found in soil [8,67,68].

The genus *Isaria* was revised from the genus *Paecilomyces* in 2005, in fact, they have similar morphological features and are very close in phylogeny [29], but many species of *Isaria* have their sexual name as *Cordyceps* [69]. *I. javanica* and *I. fumosorosea* were the very common EF species extensively used for myco-insecticide to control the aphids, whiteflies, etc., in the world. So, they should be found easily from natural environments [70,71].

Several species isolated in this research were not reported as EFs, so we carried on the bioassay experiment. *Nectria mauritiicola* and *Scopulariopsis brumptii* were found as the pathogenicity to whitefly, although their bioactivity were not very strong. If they have the potential used as biocontrol agents need further research. The other four species, *Cephalotrichum microsporum*, *Penicillium citrinum*, *Talaromyces pinophilus*, and *Umbelopsis dimorpha*, were validated for their non-pathogenicity to whitefly. Although they have not been reported as EFs, but whether they are pathogenic to more other insects needs further researches to confirm.

In conclusion, 213 EFs isolates were identified from a total of 292 isolates in the South China soil. Among them, *P. lilacinum* was absolutely the dominant EF species, while *M. anisopliae*, *I. javanica*, *B. bassiana*, *M. marquandii*, and *M. carneus* were the abundant species. The data results suggested that the Guangxi region, grass, and forest environments have the best EFs diversity. Moreover, the distribution of the EFs in the fallow land and orchard samples were decreased, which implied that the plants and insects diversity on the ground and farming activity such as fungicides spray are likely to affect the EFs diversity. Finally, it was first reported that *Nectria mauritiicola* and *Scopulariopsis brumptii* had the entomopathogenic activities to whitefly. The current research will give new insights to the understanding of EFs distribution characteristics and their biodiversities conservation.

## Figures and Tables

**Table 1:** The information of the referred fungal strains.

Species/Strain	GenBank Accession Number	Geographic Origin	Ref.
*Beauveria bassiana* ARSEF 8187	HQ444271	Canada	[24]
*Beauveria bassiana* CBS 465.70	MH859798	Israel	[25]
*Beauveria bassiana* CBS 110.25	MH854802	Sri Lanka	[25]
*Cephalotrichum microsporum* CBS 127792	MH864708	USA	[25]
*Clonostachys rosea* CBS 127881	MH864739	Spain	[25]
*Cordyceps cateniannulata* CBS 152.83	NR_111169	Thailand	[26]
*Isaria cateniannulata* ARSEF 6242	GU734760	Brazil	[27]
*Isaria farinosa* ARSEF 4029	HQ880828	Denmark	[28]
*Isaria farinosa* CBS 262.58	AY624179	Thailand	[29]
*Isaria fumosorosea* ARSEF 887	EU553334	Brazil	[30]
*Isaria fumosorosea* CBS 244.31	AY624182	Thailand	[29]
*Isaria javanica* CBS 134.22	DQ403723	USA	[31]
*Isaria javanica* CHE-CNRCB 303/2	KM234213	Mexico	[32]
*Lecanicillium psalliotae* CBS 532.81	JN049846	USA	[33]
*Metarhizium anisopliae* ARSEF 488	FJ609303	Philippines	[34]
*Metarhizium anisopliae* CBS 657.67	MH859066	New Caledonia	[25]
*Metarhizium flavoviride* CBS 218.56	MH857590	Czech	[25]
*Metarhizium flavoviride* var. *flavoviride* ARSEF 2024	AF138268	Australia	[35]
*Metarhizium marquandii* CBS 282.53	MH857200	UK	[25]
*Metarhizium marquandii* CBS 130230	MH865781	USA	[25]
*Metapochonia bulbillosa* 38G272	EU999952	Costa Rica	[36]
*Metapochonia bulbillosa* CBS 145.70	AJ292397	UK	[37]
*Metapochonia bulbillosa* FKI-4395	AB709836	Japan	[38]
*Nectria mauritiicola* P288_D1_21	JF311964	Canada	[39]
*Paecilomyces carneus* CBS 399.59	AY624170	Thailand	[29]
*Paecilomyces carneus* CG 525	EU553292	Brazil	[30]
*Paecilomyces marquandii* G28	GU566261	Czech	[40]
*Paecilomyces variotii* CBS 338.51	FJ389930	Netherlands	[41]
*Penicillium citrinum* CBS 117.64	MH858380	Netherlands	[25]
*Phialophora verrucosa* CBS 286.47	KF928455	Netherlands	[42]
*Pochonia chlamydosporia* var. *catenulata* CBS 504.66	MH858871	Canada	[25]
*Pochonia chlamydosporia* CBS 103.65	AJ292397	UK	[37]
*Pochonia chlamydosporia* CBS 429.64	MH858477	Brazil	[25]
*Purpureocillium lilacinum* ATT161	HQ607867	USA	[43]
*Purpureocillium lilacinum* CBS284.36	NR_111432	Spain	[26]
*Purpureocillium lilacinum* NRRL 6453	HQ842836	Netherlands.	[44]
*Schizophyllum commune* CBS 124811	MH863418	Netherlands	[25]
*Scopulariopsis brumptii* CBS 121662	LN850803	USA	[45]
*Simplicillium lanosoniveum* CBS 531.72	MH860557	USA	[25]
*Talaromyces pinophilus* CBS 631.66	NR_111691	Netherlands	[26]
*Umbelopsis dimorpha* CBS 110039	KC489478	New Zealand	[46]

**Table 2:** The fungi isolation and biodiversity of different regions.

Region	Sample Numbers			Isolation Rate (%)		Isolate Number		EF Species	Shannon Wiener Index
	Total	Fungi	EFs	Fungi	EFs	Total	EFs		
Fujian	26	21	21	80.77	80.77	27	27	6	1.43
Guangdong	78	68	55	87.18	70.51	85	68	10	1.86
Guangxi	75	69	56	92.00	74.67	160	99	18	2.29
Hainan	19	18	18	94.74	94.74	20	19	4	0.73
Total	198	176	150	88.89 *	75.76 *	292	213	19	

* The means of isolation rate (%) in all regions. EFs: Entomopathogenic fungi.

**Table 3:** The fungi isolation and biodiversity of different samples.

Sample Vegetation	Sample Numbers			Isolation Rate (%)		Isolate Number		EF Species	Shannon–Wiener Index
	Total	Fungi	EFs	Fungi	EFs	Total	EFs		
Crop	48	43	41	89.58	85.42	58	51	11	1.89
Eucalyptus	30	23	19	76.67	63.33	41	26	9	1.93
Fallowland	32	28	25	87.50	78.13	37	32	7	1.52
Forest	32	28	24	87.50	75.00	53	38	12	1.97
Grass	44	42	31	95.45	70.45	83	53	14	2.14
Orchard	12	12	10	100	83.33	20	13	5	1.52
Total	198	176	150	88.89 *	75.76 *	292	213	19	

* The means of isolation rate (%) in all sample vegetation.

**Table 4:** The pathogenicities of fungal isolates against the second nymphs of the B-biotype whitefly.

Isolate	Species	Accumulated Mortality (%) * on Post-Treatment Days			
		4 Days	6 Days	8 Days	10 Days
BbGD07	*Beauveria bassiana*	27.64 ± 5.32 a	33.67 ± 4.69 a	39.9 ± 5.48 a	55.56 ± 7.89 c
IfFJ05	*Isaria javanica*	6.94 ± 2.13 d	17.87 ± 2.23 c	42.68 ± 1.87 a	61.03 ± 3.08 a
LpGD62	*Lecanicillium psalliotae*	8.19 ± 3.15 c	12.33 ± 1.96 c	39.40 ± 4.32 a	66.19 ± 8.25 a
ClspGX21G03	*Clonostachys* sp.	12.67 ± 3.06 b	22.17 ± 2.34 b	35.06 ± 3.16 b	44.04 ± 4.15 d
MaFJ07	*Metarhizium anisopliae*	8.15 ± 3.18 c	13.11 ± 4.98 c	44.41 ± 8.08 a	61.02 ± 5.33 a
NmGX2905	*Nectria mauritiicola*	6.00 ± 2.00 d	16.75 ± 4.06 c	27.46 ± 5.48 c	35.75 ± 7.47 e
PamGD01	*Paecilomyces marquandii*	8.31 ± 3.22 c	20.88 ± 6.48 b	46.53 ± 0.42 a	65.97 ± 2.99 a
PhvGX77A02	*Phialophora verrucosa*	9.00 ± 3.83 c	16.75 ± 3.71 c	28.5 ± 5.21 c	38.34 ± 5.45 e
PlHN01	*Purpureocillium lilacinum*	10.26 ± 3.37 bc	16.46 ± 2.71 c	40.29 ± 3.13 a	58.69 ± 3.51 b
PocGD43	*Pochonia chlamydosporia*	7.89 ± 2.56 cd	15.75 ± 1.38 c	44.78 ± 2.01 a	64.40 ± 1.87 a
SbGX7705	*Scopulariopsis brumptii*	8.00 ± 3.65 c	14.72 ± 3.71 c	22.8 ± 4.27 c	29.02 ± 3.54 f
CmGX11G02	*Cephalotrichum microsporum*	1.32 ± 0.45 e	3.15 ± 0.46 d	4.25 ± 0.39 d	4.75 ± 0.40 g
PecGX1605	*Penicillium citrinum*	1.18 ± 0.45 e	2.57 ± 0.41 d	3.79 ± 0.32 d	4.11 ± 0.33 g
TpGX05A01	*Talaromyces pinophilus*	1.26 ± 0.31 e	2.67 ± 0.37 d	3.91 ± 0.38 d	4.08 ± 0.34 g
UdGX13S05	*Umbelopsis dimorpha*	1.31 ± 0.42 e	2.78 ± 0.41 d	3.79 ± 0.32 d	3.92 ± 0.31 g
Control		1.25 ± 0.55 e	2.50 ± 0.50 d	3.50 ± 0.41 d	3.75 ± 0.38 g

* The mean ± SE at the days post-treatment, the different letters behind indicate the significant difference (*p* < 0.05) by DMRT (Duncan’s multiple range test).

## Supplementary Materials

The following are available online at https://www.mdpi.com/2076-2607/7/9/311/s1, Table S1: Information of the sites soil samples collected and fungi isolation and identification.
