# Supplementary material for: Biodiversity of Entomopathogenic Fungi in the Soils of South China
Source: Microorganisms. 2019 Sep 3;7(9):311. doi: 10.3390/microorganisms7090311 (PMC6780753; doi:10.3390/microorganisms7090311)
Supplement: Supplementary file 1 [file microorganisms-07-00311-s001.pdf]

Table S1. The information of soil samples collection and fungal isolation.

NO.	Address	Site: Longitude and Latitude	Site: Sample Environment	Isolate	Genebank Access NO.	Species
FJ01	Nanping, Fujian	E118.18,N26.64	Crop	BbFJ01	MH483616	*Beauveria bassiana*
				IfFJ01	MH483617	*Isaria javanica*
FJ02	Liancheng, Fujian	E116.78,N25.71	Fallowland	IfFJ02	MH483619	*Isaria javanica*
				PlFJ02	MH483618	*Purpureocillium lilacinum*
FJ03	Nanping, Fujian	E117.77,N26.80	Crop	IjFJ03	MH483621	*Isaria javanica*
				PlFJ03	MH483620	*Purpureocillium lilacinum*
FJ04	Fuan, Fujian	E119.65,N27.07	Fallowland	IfFJ04	MH483623	*Isaria javanica*
				PlFJ04	MH483622	*Purpureocillium lilacinum*
FJ05	Shaxian, Fujian	E117.97,N26.50	Crop	IfFJ05	MH483624	*Isaria javanica*
FJ06	Wuyishan, Fujian	E117.99,N27.61	Crop	IfFJ06	MH483625	*Isaria javanica*
FJ07	Wuping, Fujian	E116.28,N24.94	Grassy	MaFJ07	MH483626	*Metarhizium anisopliae*
FJ08	Xiapu, Fujian	E120.01,N26.89	Crop	PlFJ08	MH483627	*Purpureocillium lilacinum*
FJ09	Huian, Fujian	E118.76,N24.92	Crop	PlFJ09	MH483628	*Purpureocillium lilacinum*
				PamFJ09	MH483629	*Metarhizium marquandii*
FJ10	Jiangle, Fujian	E117.40,N26.73	Fallowland	PlFJ10	MH483630	*Purpureocillium lilacinum*
FJ11	Fuding, Fujian	E119.65,N27.07	Fallowland	BbFJ11	MH483631	*Beauveria bassiana*
FJ12	Zhenghe, Fujian	E119.18,N27.16	Fallowland	PlFJ12	MH483632	*Purpureocillium lilacinum*
FJ13	Pucheng, Fujian	E118.37,N27.70	Crop			
FJ14	Luoyuan, Fujian	E119.50,N26.47	Crop	PlFJ14	MH483633	*Purpureocillium lilacinum*
FJ15	Lianjiang, Fujian	E119.55,N26.20,	Fallowland	PlFJ15	MH483634	*Purpureocillium lilacinum*
FJ16	Minhou, Fujian	E119.33,N25.86	Crop	PobFJ16	MH483635	*Metapochonia bulbillosa*
				PlFJ16	MH483636	*Purpureocillium lilacinum*
FJ17	Yongan, Fujian	E117.32,N25.96	Fallowland	IfFJ17	MH483637	*Isaria javanica*
FJ18	Yanping, Fujian	E118.01,N26.59	Fallowland			
FJ19	Quanzhou, Fujian	E118.20,N26.64	Fallowland			
FJ20	Shanghang, Fujian	E116.57,N25.12	Fallowland	PlFJ20	MH483638	*Purpureocillium lilacinum*
FJ21	Shaowu, Fujian	E117.48,N27.28	Grassy			
FJ22	Zhangpu, Fujian	E117.59, N23.77	Fallowland	PlFJ22	MH483639	*Purpureocillium lilacinum*
FJ23	Zhaoan, Fujian	E117.24, N23.71	Crop	BbFJ23	MH483640	*Beauveria bassiana*
FJ24	Zherong, Fujian	E119.83,N27.22	Crop	BbFJ24	MH483641	*Beauveria bassiana*
FJ25	Taining, Fujian	E117.13,N26.87	Fallowland	MaFJ25	MH483642	*Metarhizium anisopliae*
FJ26	Xiamen, Fujian	E118.20,N24.69	Fallowland			
GD01	Boluo, Guangdong	E114.56,N23.54	Fallowland	PamGD01	MH483643	*Metarhizium marquandii*
GD02	Yaoping, Guangdong	E116.80,N24.13	Crop	IfGD02	MH483644	*Isaria javanica*
GD03	Jiaoling, Guangdong	E116.37,N24.84	Crop	BbGD03	MH483645	*Beauveria bassiana*
GD04	Meixian, Guangdong	E116.15,N24.43	Crop	BbGD04	MH483646	*Beauveria bassiana*
GD05	Xingning, Guangdong	E115.78,N24.11	Crop	BbGD05	MH483647	*Beauveria bassiana*
GD06	Wuhua, Guangdong	E115.44,N24.07	Crop	BbGD06	MH483648	*Beauveria bassiana*
GD07	Xuwun, Guangdong	E110.25,N20.42	Crop	BbGD07	MH483649	*Beauveria bassiana*
				IfGD07	MH483651	*Isaria javanica*
				PlGD07	MH483650	*Purpureocillium lilacinum*
GD08	Nansha, Guangdong	E113.58,N22.72	crop	BbGD08	MH483652	*Beauveria bassiana*
GD09	Conghua, Guangdong	E113.68,N23.66	Forest	GD09		
GD10	Suixi, Guangdong	E110.03,N21.27	Fallowland	BbGD10	MH483654	*Beauveria bassiana*
				PlGD10	MH483653	*Purpureocillium lilacinum*

GD11	Leizhou, Guangdong	E110.01,N20.79	Forest	PlGD11	MH483655	*Purpureocillium lilacinum*
				IfGD12	MH483657	*Isaria javanica*
GD12	Meixian, Guangdong	E116.40,N24.65	Fallowland	PlGD12	MH483656	*Purpureocillium lilacinum*
GD13	Dongyuan, Guangdong	E114.75,N23.82	Fallowland	BbGD13	MH483658	*Beauveria bassiana*
GD14	Enping, Guangdong	E112.39,N22.30	Fallowland	IfGD14	MH483659	*Isaria javanica*
GD15	Huizhou, Guangdong	E114.03, N23.60	Fallowland	IfGD15	MH483661	*Isaria javanica*
				PlGD15	MH483660	*Purpureocillium lilacinum*
GD16	Jiedong, Guangdong	E116.36,N23.60	Crop			
GD17	Boluo, Guangdong	E114.28,N23.35	Crop	IfGD17	MH483662	*Isaria javanica*
GD18	Longmen, Guangdong	E113.98,N23.53	Fallowland	IfGD18	MH483663	*Isaria javanica*
GD19	Huidong, Guangdong	E114.95,N22.05	Crop	BbGD19	MH483665	*Beauveria bassiana*
				PspGD19	MH483664	
GD20	Longmen, Guangdong	E114.13,N23.66	Orchard	BbGD20	MH483667	*Beauveria bassiana*
				IfGD20	MH483668	*Isaria javanica*
				PspGD20	MH483666	
GD21	Zhongshan, Guangdong	E113.44,N22.36	Crop	IfGD21	MH483669	*Isaria javanica*
GD22	Taishan, Guangdong	E112.80,N21.70	Crop	PlGD22	MH483670	*Purpureocillium lilacinum*
GD23	Enping, Guangdong	E112.35,N21.94	Crop	PacGD23	MH483672	*Metarhizium carneum*
				PlGD23	MH483671	*Purpureocillium lilacinum*
GD24	Shihui, Guangdong	E112.68,N23.43	Eucalyptus			
GD25	Yangchun, Guangdong	E111.69,N22.22	Orchard	MaGD25	MH483674	
				PacGD25	MH483673	*Metarhizium carneum*
GD26	Yangchun, Guangdong	E111.87,N22.30	Forest	PacGD26	MH483675	*Metarhizium carneum*
GD27	Dianbai, Guangdong	E111.35,N21.64	Crop	PlGD27	MH483676	*Purpureocillium lilacinum*
GD28	Gaozhou, Guangdong	E110.97,N22.03	Orchard	ScGD28	MH483677	*Schizophyllum commune*
GD29	Yuncheng, Guangdong	E112.26,N22.93	Grassy	PacGD29	MH483678	*Metarhizium carneum*
				PlGD29	MH483679	*Purpureocillium lilacinum*
GD30	Yunan, Guangdong	E111.61,N23.04	Crop	PlGD30	MH483680	*Purpureocillium lilacinum*
GD31	Luoding, Guangdong	E111.46,N22.56	Eucalyptus			
GD32	Yunfuo, Guangdong	E111.74,N23.00	Grassy	MaGD32	MH483681	*Metarhizium anisopliae*
GD33	Gaoyao, Guangdong	E112.76,N23.09	Forest	MaGD33	MH483682	
GD34	Guangning, Guangdong	E112.42,N23.55	Forest	PlGD34	MH483683	*Purpureocillium lilacinum*
GD35	Ruyuan, Guangdong	E113.14,N24.54	Grassy	PacGD35	MH483684	*Metarhizium carneum*
GD36	Gaoyao, Guangdong	E112.68,N22.97	Eucalyptus	PacGD36	MH483685	*Metarhizium carneum*
GD37	Huaiji, Guangdong	E112.19,N23.87	Eucalyptus	PacGD37	MH483686	*Metarhizium carneum*
GD38	Jingmen, Guangdong	E112.84,N22.37	Eucalyptus			
GD39	Lianshan, Guangdong	E112.10,N24.51	Grassy	MaGD39	MH483687	*Metarhizium anisopliae*
GD40	Liannan, Guangdong	E113.04,N23.70	Forest	PlGD40	MH483688	*Purpureocillium lilacinum*
GD41	Lianzhou, Guangdong	E112.37,N24.65	Orchard	PacGD41	MH483689	*Metarhizium carneum*
GD42	Yangshan, Guangdong	E112.68,N24.44	Forest	MaGD42	MH483690	*Metarhizium anisopliae*
GD43	Yingde, Guangdong	E112.89,N24.39	Grassy	PocGD43	MH483691	*Pochonia chlamydosporia*
GD44	Luoding, Guangdong	E111.82,N22.77	Grassy	PlGD44	MH483692	*Purpureocillium lilacinum*
GD45	Lianping, Guangdong	E114.42,N24.39	Fallowland	PacGD45	MH483693	*Metarhizium carneum*
GD46	Yingde, Guangdong	E113.04,N24.43	Forest	MaGD46	MH483695	*Metarhizium anisopliae*
				PocGD46	MH483694	*Pochonia chlamydosporia*
GD47	Shixing, Guangdong	E114.06,N24.82	Forest	GD47		
GD48	Meixian, Guangdong	E116.41,N24.65	Fallowland	BbGD48	MH483696	*Beauveria bassiana*
				PlGD48	MH483697	*Purpureocillium lilacinum*
GD49	Ruyuan, Guangdong	E113.11,N24.81	Grassy	PacGD49	MH483698	*Metarhizium carneum*
GD50	Nanxiang, Guangdong	E119.38,N25.20	Forest	PlGD50	MH483699	*Purpureocillium lilacinum*
GD51	Heping, Guangdong	E114.90,N24.27	Grassy	GD51		

GD52	Shixing, Guangdong	E114.10,N24.65	Forest	PlGD52	MH483700	*Purpureocillium lilacinum*
GD53	Ruyuan, Guangdong	E113.08,N24.57	Forest			
GD54	Lechang, Guangdong	E113.34,N25.09	Grassy	PobGD54	MH483702	*Metapochonia bulbillosa*
				PlGD54	MH483701	*Purpureocillium lilacinum*
GD55	Wuchuan, Guangdong	E110.59,N21.36	Forest			
GD56	Lianjing, Guangdong	E110.14,N21.65	Forest	MaGD56	MH483704	*Metarhizium anisopliae*
				PlGD56	MH483703	*Purpureocillium lilacinum*
GD57	Zijing, Guangdong	E114.71,N23.52	Eucalyptus			
GD58	Shixing, Guangdong	E114.07,N24.81	Grassy	MaGD58	MH483706	*Metarhizium anisopliae*
				PlGD58	MH483705	*Purpureocillium lilacinum*
GD59	Suixi, Guangdong	E110.09,N21.18	Forest	PlGD59	MH483707	*Purpureocillium lilacinum*
GD60	Yangxi, Guangdong	E111.62,N21.74	Crop	PlGD60	MH483708	*Purpureocillium lilacinum*
GD61	Enping, Guangdong	E112.30,N22.24	Grassy	PlGD61	MH483709	*Purpureocillium lilacinum*
GD62	Puning, Guangdong	E116.18,N23.38	Crop	LpGD62	MH483710	*Lecanicillium psalliotae*
GD63	Fogang, Guangdong	E113.48,N23.74	Crop	BbGD63	MH483711	*Beauveria bassiana*
GD64	Fengkai, Guangdong	E111.49,N23.30	Grassy	GD64		
GD65	Wengyuan, Guangdong	E114.23,N 24.42	Grassy	SlGD65	MH483712	*Simplicillium lanosoniveum*
GD66	Lianshan, Guangdong	E112.10,N24.52	Forest			
GD67	Lianping, Guangdong	E114.40,N24.38	Fallowland	BbGD67	MH483713	*Beauveria bassiana*
GD68	Lianping, Guangdong	E114.78,N24.17	Eucalyptus	GD68		
GD69	Shanwei, Guangdong	E115.84,N22.95	Fallowland	GD69		
GD70	Maoming, Guangdong	E110.98,N22.57	Grassy	GD70		
GD71	Gaoyao, Guangdong	E112.47,N23.00	Grassy	GD7101		
				GD7102		
GD72	Heshan, Guangdong	E113.03,N22.78	Grassy	GD72		
GD73	Taishan, Guangdong	E112.81,N22.37	Crop			
GD74	Kaiping, Guangdong	E112.77,N22.45	Crop	MaGD74	MH483714	*Metarhizium anisopliae*
GD75	Taishan, Guangdong	E112.80,N21.97	Crop			
GD76	Jinagcheng, Guangdong	E111.98,N21.90	Grassy	GD76		
	Huazhou, Guangdong	E110.56,N21.93	Crop	PamGD77	MH483715	*Metarhizium marquandii*
GD78	Dianbai, Guangdong	E111.16,N21.48	Forest	GD78		
GX01	Cengxi, Guangdong	E111.22,N22.9	Fallowland	GX0101		
GX02	Cengxi, Guangdong	E110.91,N22.91	Eucalyptus	MaGX02A02	MH483826	*Metarhizium anisopliae*
				GX02A01		
			Grassy	PlGX0201	MH483825	*Purpureocillium lilacinum*
				GX0202		
GX03	Rongxian, Guangdong	E110.56,N22.9	Eucalyptus	GX03A01		
			Grassy	GX0301		
				GX0302		
GX04	Beiliu, Guangdong	E110.32,N22.78	Eucalyptus	PlGX04A01	MH483828	*Purpureocillium lilacinum*
			Forest	PlGX0401	MH483827	*Purpureocillium lilacinum*
GX05	Fumian, Guangdong	E110.05,N22.37	Eucalyptus	TpGX05A01	MH483830	*Talaromyces pinophilus*
			Forest	PlGX0501	MH483829	*Purpureocillium lilacinum*
GX06	Bobai, Guangdong	E109.74,N21.83	Eucalyptus	PlGX06A01	MH483833	*Purpureocillium lilacinum*
			Grassy	MaGX0601	MH483831	*Metarhizium anisopliae*
				MfGX0603	MH483832	*Metarhizium flavoviride*
				GX0602		
				GX0604		
GX07	Hepu, Guangdong	E109.24,N21.66	Eucalyptus			
GX08	Qinnan, Guangdong	E108.65,N21.89	Eucalyptus	PocGX08A01	MH483836	*Pochonia chlamydosporia*
			Grassy	PobGX0801	MH483834	*Metapochonia bulbillosa*

				PlGX0802	MH483835	*Purpureocillium lilacinum*
GX09	Shangsi, Guangdong	E107.97,N22.12	Eucalyptus	MaGX09A02	MH483837	*Metarhizium anisopliae*
				GX09A01		
			Grassy	GX0901		
GX10	Fusuisui, Guangdong	E107.7,N22.28	Eucalyptus	BbGX10A01	MH483840	*Beauveria bassiana*
			Grassy	PamGX1002	MH483839	*Metarhizium marquandii*
				PlGX1001	MH483838	*Purpureocillium lilacinum*
GX11	Daxing, Guangdong	E107.06,N22.81	Crop	CmGX11G02	MH483842	*Cephalotrichum microsporum*
				GX11G01		
			Grassy	MgGX1103	MH483841	*Metarhizium anisopliae*
				GX1101		
				GX1102		
GX12	Jingxi, Guangdong	E106.61,N23	Crop	PamGX12Y01	MH483843	*Metarhizium marquandii*
			Fallowland	GX1201		
GX13	Debao, Guangdong	E106.64,N23.29	Forest	MaGX13S01	MH483844	*Metarhizium anisopliae*
				UdGX13S05	MH483846	*Umbelopsis dimorpha*
				PobGX13S04	MH483845	*Metapochonia bulbillosa*
				GX13S02		
				GX13S03		
			Grassy	PocGX13Z02	MH483848	*Pochonia chlamydosporia*
				PlGX13Z01	MH483847	*Purpureocillium lilacinum*
GX14	Youjiang, Guangdong	E106.63,N23.96	Eucalyptus			
			Forest	PamGX1402	MH483850	*Metarhizium marquandii*
				PlGX1401	MH483849	*Purpureocillium lilacinum*
GX15	Youjiang, Guangdong	E106.33,N24.2	Grassy	GX1501		
				GX1502		
			Fallowland			
			Forest	MaGX16S01	MH483855	*Metarhizium anisopliae*
				PobGX16S04	MH483857	*Metapochonia bulbillosa*
				PlGX16S03	MH483856	*Purpureocillium lilacinum*
				GX16S02		
GX16	Tiandong, Guangdong	E107.03,N23.61	Grassy	MaGX1602	MH483851	*Metarhizium anisopliae*
				PamGX1603	MH483852	*Metarhizium marquandii*
				PecGX1605	MH483854	*Penicillium citrinum*
				PobGX1604	MH483853	*Metapochonia bulbillosa*
				GX1601		
GX17	Pingguo, Guangdong	E107.56,N23.28	Eucalyptus	PamGX17A02	MH483861	*Metarhizium marquandii*
				PlGX17A01	MH483860	*Purpureocillium lilacinum*
			Grassy	MaGX1701	MH483858	*Metarhizium anisopliae*
				PlGX1702	MH483859	*Purpureocillium lilacinum*
GX18	Pingguo, Guangdong	E107.7,N23.43	Crop			
			Forest	MaGX18S03	MH483862	*Metarhizium anisopliae*
				GX18S01		
				GX18S02		
GX19	Wuming, Guangdong	E108.21,N23.43	Orchard	MaGX19J01	MH483863	*Metarhizium anisopliae*
				GX19J02		
				MaGX19S02	MH483865	*Metarhizium anisopliae*
				PlGX19S01	MH483864	*Purpureocillium lilacinum*
GX20	Wuming, Guangdong	E108.27,N23.01	Eucalyptus	IfGX20A02	MH483866	*Isaria javanica*
				GX20A01		
			Forest	IfGX2002	MH483867	*Isaria javanica*
				GX2001		
GX21	Qingxiu, Guangdong	E108.77,N22.83	Crop	ClspGX21G03	MH430067	*Clonostachys* sp.
				IfGX21G01	MH483868	*Isaria javanica*
				GX21G02		

			Orchard	IfGX21L01	MH483869	*Isaria javanica*
				PecGX21L02	MH483870	*Penicillium citrinum*
				GX21L03		
GX22	Hengxian, Guangdong	E109.23,N22.87	Eucalyptus	BbGX22A02	MH483871	*Beauveria bassiana*
				GX22A01		
			Grassy	GX2201		
				GX2202		
GX23	Gangnan, Guangdong	E109.72,N23	Eucalyptus	MaGX23A01	MH483874	*Metarhizium anisopliae*
				GX23A02		
			Grassy	IfGX2303	MH483873	*Isaria javanica*
				PocGX2301	MH483872	*Pochonia chlamydosporia*
				GX2302		
GX24	Xuanwu, Guangdong	E109.71,N23.59	Crop	PamGX24G02	MH483875	*Metarhizium marquandii*
				GX24G01		
GX25	Xiangzhou, Guangdong	E109.59,N23.93	Eucalyptus	GX25A01		
			Grassy	GX2501		
GX26	Liujiang, Guangdong	E109.28,N24.25	Grassy	IfGX26S01	MH483876	*Isaria javanica*
				GX26G01		
				GX26S02		
GX27	Liucheng, Guangdong	E109.28,N24.65	Eucalyptus	IfGX2701	MH483877	*Isaria javanica*
				GX2702		
GX28	Rongshui, Guangdong	E109.28,N25.06	Grassy	IfGX2802	MH483878	*Isaria javanica*
				GX2801		
GX29	Liucheng, Guangdong	E108.95,N24.51	Grassy	IfGX2906	MH483880	*Isaria javanica*
				LspGX2904	MH483879	*Lecanicillium psalliotae*
				NmGX2905	MH430069	*Nectria mauritiicola*
				PamGX2907	MH483881	*Metarhizium marquandii*
				GX2901		
				GX2902		
				GX2903		
GX30	Yizhou, Guangdong	E108.31,N24.68	Eucalyptus	IfGX30A01	MH483882	*Isaria javanica*
				GX30A02		
			Orchard	IfGX3001	MH483883	*Isaria javanica*
GX31	Jingchengjiang, Guangdong	E107.87,N24.68	Crop	TpGX3101	MH483884	*Talaromyces pinophilus*
GX32	Jingchengjiang, Guangdong	E107.77,N24.84	Eucalyptus	IfGX32A03	MH483885	*Isaria javanica*
				GX32A01		
				GX32A02		
			Forest	IcGX32S01	MH483886	*Isaria cateniannulata*
				PobGX32S02	MH483887	*Metapochonia bulbillosa*
GX33	Nandan, Guangdong	E107.56,N25.04	Crop	MfGX33Y01	MH483888	*Metarhizium flavoviride*
				PocGX33Y03	MH483889	*Pochonia chlamydosporia*
				GX33Y02		
			Fallowland	IfGX33H05	MH483892	*Isaria fumosorosea*
				MaGX33H02	MH483890	*Metarhizium anisopliae*
				PamGX33H03	MH483891	*Metarhizium marquandii*
				GX33H01		
				GX33H04		
GX65	Quanzhou, Guangdong	E110.94,N26.29	Forest	BbGX6502	MH483894	*Beauveria bassiana*
				MfGX6501	MH483893	*Metarhizium flavoviride*
				PamGX6506	MH483897	*Metarhizium marquandii*
				PacGX6505	MH483896	*Metarhizium carneum*
				PlGX6503	MH483895	*Purpureocillium lavendulum*
				GX6504		
GX66	Quanzhou, Guangdong	E111.01,N26.23	Forest	MfGX6601	MH483898	*Metarhizium flavoviride*
				GX6602		
				GX6603		
GX67	Xingan, Guangdong	E110.77,N25.71	Forest	IfaGX6701	MH483899	*Isaria farinose*
GX68	Lingchuan, Guangdong	E110.31,N25.41	Grassy	PlaGX6801	MH483900	*Purpureocillium lavendulum*
				PlGX6802	MH483901	*Purpureocillium lilacinum*

GX69	Yongfu, Guangdong	E110,N24.99	Grassy	PlGX6901	MH483902	*Purpureocillium lilacinum*
				GX6902		
GX70	Luzhai, Guangdong	E109.84,N24.51	Eucalyptus	BbGX70A02	MH483907	*Beauveria bassiana*
				IfGX70A01	MH483906	*Isaria fumosorosea*
				PlGX70A03	MH483908	*Purpureocillium lilacinum*
			Grassy	MaGX7002	MH483904	*Metarhizium anisopliae*
				PamGX7001	MH483903	*Metarhizium marquandii*
				PlGX7003	MH483905	*Purpureocillium lilacinum*
GX71	Luzhai, Guangdong	E110.01,N24.36	Forest	PlGX7101	MH483909	*Purpureocillium lilacinum*
				GX7102		
GX72	Jingxiu, Guangdong	E110.09,N24.35	Eucalyptus	MaGX7201	MH483910	*Metarhizium anisopliae*
				PlGX7202	MH483911	*Purpureocillium lilacinum*
GX73	Lipu, Guangdong	E110.36,N24.48	Forest	BbGX7303	MH483914	*Beauveria bassiana*
				PamGX7301	MH483912	*Metarhizium marquandii*
				PlGX7302	MH483913	*Purpureocillium lilacinum*
GX74	Pingle, Guangdong	E110.62,N24.61	Orchard	PlGX7402	MH483915	*Purpureocillium lilacinum*
				GX7401		
GX75	Pingle, Guangdong	E110.8,N24.66	Forest			
GX76	Zhongshan, Guangdong	E111.09,N24.46	Grassy			
GX77	Pinggui, Guangdong	E111.58,N24.37	Eucalyptus	BbGX77A01	MH483921	*Beauveria bassiana*
				PlGX77A03	MH483922	*Purpureocillium lilacinum*
				PhvGX77A02	MH430070	*Phialophora verrucosa*
				PlGX77A04	MH483923	*Purpureocillium lilacinum*
			Grassy	BbGX7703	MH483918	*Beauveria bassiana*
				IfGX7704	MH483919	*Isaria javanica*
				MaGX7702	MH483917	*Metarhizium anisopliae*
				PamGX7701	MH483916	*Metarhizium marquandii*
				PlGX7706	MH483920	*Purpureocillium lilacinum*
				SbGX7705	MH430069	*Scopulariopsis brumptii*
GX78	Babu, Guangdong	E111.73,N24.05	Eucalyptus	PlGX78A01	MH483925	*Purpureocillium lilacinum*
				PecGX78A02	MH483926	*Penicillium citrinum*
			Grassy	PlGX7801	MH483924	*Purpureocillium lilacinum*
HN01	Dongfang, Hainan	E108.67,N19.11	Crop	PlHN01	MH483716	*Purpureocillium lilacinum*
HN02	Qiongzhong, Hainan	E109.86,N19.08	Grassy	PlHN02	MH483717	*Purpureocillium lilacinum*
HN03	Chengmai, Hainan	E110.02,N19.74	Crop	PlHN03	MH483718	*Purpureocillium lilacinum*
HN04	Anding, Hainan	E110.45,N19.48	Orchard	PlHN04	MH483719	*Purpureocillium lilacinum*
HN05	Changjiang, Hainan	E109.06,N19.28	Crop	BbHN05	MH483720	*Beauveria bassiana*
				PspHN05	MH483721	
HN06	Lingshui, Hainan	E110.19,N18.57	Fallowland	PlHN06	MH483722	*Purpureocillium lilacinum*
HN07	Tuenchang, Hainan	E110.09,N19.40	Orchard	PlHN07	MH483723	*Purpureocillium lilacinum*
HN08	Qionghai, Hainan	E110.49,N19.12	Orchard	MaHN08	MH483724	*Metarhizium anisopliae*
HN09	Wuzhishan, Hainan	E109.55,N18.75	Crop	PlHN09	MH483725	*Purpureocillium lilacinum*
HN10	Wenchang, Hainan	E110.66,N19.57	Forest	PlHN10	MH483726	*Purpureocillium lilacinum*
HN11	Wanning, Hainan	E110.31,N18.72	Orchard			

HN12	Shanya, Hainan	E109.56,N18.27	Fallowland	PlHN12	MH483727	*Purpureocillium lilacinum*
HN13	Ledong, Hainan	E108.89,N18.47	Crop	PlHN13	MH483728	*Purpureocillium lilacinum*
HN14	Haikou, Hainan	E110.27,N19.89	Fallowland	PlHN14	MH483729	*Purpureocillium lilacinum*
HN15	Zhagzhou, Hainan	E109.46,N19.48	Crop	BbHN15	MH483730	*Beauveria bassiana*
HN16	Baisha, Hainan	E109.18,N19.41	Fallowland	PlHN16	MH483731	*Purpureocillium lilacinum*
HN17	Baotin, Hainan	E109.67,N18.40	Grassy	PlHN17	MH483732	*Purpureocillium lilacinum*
HN18	Linggao, Hainan	E109.76,N19.77	Crop	PlHN18	MH483733	*Purpureocillium lilacinum*
HN19	Baotin, Hainan	E109.62,N18.61	Crop	PspHN19	MH483735	*Purpureocillium lilacinum*
				PocHN19	MH483734	*Pochonia chlamydosporia*
